# Genome-Wide Association Studies Identified Three Independent Polymorphisms Associated with α-Tocopherol Content in Maize Kernels

**DOI:** 10.1371/journal.pone.0036807

**Published:** 2012-05-15

**Authors:** Qing Li, Xiaohong Yang, Shutu Xu, Ye Cai, Dalong Zhang, Yingjia Han, Lin Li, Zuxin Zhang, Shibin Gao, Jiansheng Li, Jianbing Yan

**Affiliations:** 1 National Maize Improvement Center of China, China Agricultural University, Beijing, China; 2 National Key Laboratory of Crop Improvement, Huazhong Agricultural University, Wuhan, Hubei, China; 3 Maize Research Institute, Sichuan Agricultural University, Ya’an, Sichuan, China; Nanjing Agricultural University, China

## Abstract

Tocopherols are a class of four natural compounds that can provide nutrition and function as antioxidant in both plants and animals. Maize kernels have low α-tocopherol content, the compound with the highest vitamin E activity, thus, raising the risk of vitamin E deficiency in human populations relying on maize as their primary vitamin E source. In this study, two insertion/deletions (InDels) within a gene encoding γ-tocopherol methyltransferase, *Zea mays VTE4* (*ZmVTE4*), and a single nucleotide polymorphism (SNP) located ∼85 kb upstream of *ZmVTE4* were identified to be significantly associated with α-tocopherol levels in maize kernels by conducting an association study with a panel of ∼500 diverse inbred lines. Linkage analysis in three populations that segregated at either one of these three polymorphisms but not at the other two suggested that the three polymorphisms could affect α-tocopherol content independently. Furthermore, we found that haplotypes of the two InDels could explain ∼33% of α-tocopherol variation in the association panel, suggesting *ZmVTE4* is a major gene involved in natural phenotypic variation of α-tocopherol. One of the two InDels is located within the promoter region and associates with *ZmVTE4* transcript level. This information can not only help in understanding the underlying mechanism of natural tocopherol variations in maize kernels, but also provide valuable markers for marker-assisted breeding of α-tocopherol content in maize kernels, which will then facilitate the improvement of maize as a better source of daily vitamin E nutrition.

## Introduction

Tocopherols are lipid-soluble antioxidants that occur in four natural forms: α-tocopherol (αT), γ-tocopherol (γT), δ-tocopherol (δT) and β-tocopherol (βT) [1]. Among all the tocopherols, αT has the highest vitamin E activity and is preferentially bound by the hepatic αT transfer protein in humans [2]. Sufficient αT intake is very important for human because it can help to improve immune responsiveness and protect cells against oxidative stresses [3]–[5]. However, it is estimated that over 20% of the examined people in both developed and developing countries has suboptimal plasma αT levels [6]–[9]. While vitamin E supplementation is an affordable way for people in developed countries, it is usually unavailable for people in developing countries. Thus, biofortification of vitamin E in food may represent an economic and efficient way to ensure vitamin E intake in developing countries. Tocopherols are also indispensable for fetal development in rats, and sufficient intake of tocopherols can improve lipid stability in steaks, thus, tocopherol supplementation has become a routine procedure to promote the growth of farm animals [10], [11]. Tocopherols also play an important role in a range of plant processes, such as seed maturation, storage and germination, photo-assimilates transportation and abiotic stress response [12]–[15].

**Figure 1:**
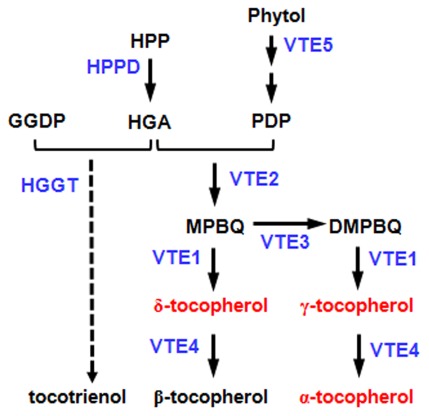
Simplified tocopherol biosynthetic pathway [1], [16], [17]. The three tocopherol compounds measured in this study (red) and the key genes involved in this process (blue) are shown. DMPBQ, 2,3-dimethyl-5-phytyl-1,4-benzoquinone; GGDP, geranylgeranyl diphosphate; HGA, homogentisic acid; HGGT, homogentisic acid geranylgeranyl transferase; HPP, p-hydroxyphenylpyruvate; HPPD, HPP dioxygenase; MPBQ, 2-methyl-6-phytyl-1,4-benzoquinone; PDP, phytyl-diphosphate; VTE1, tocopherol cyclase; VTE2, homogentisate phytyltransferase; VTE3, MPBQ methyltransferase; VTE4, tocopherol methyltransferase; VTE5, phytol kinase.

In view of the importance of tocopherols in animals and plants, the key genes involved in tocopherol biosynthesis have been elucidated in *Arabidopsis* and other model organisms (Figure 1) [1], [16], [17]. A quantitative trait locus (QTL) study performed in *Arabidopsis* suggested that some of these key genes and a few additional loci contribute to natural tocopherol variations [18]. Identifying such genetic variations in other plants, especially in important crop plants, can not only better our understanding of the genetic mechanisms controlling tocopherol variations, but also provide invaluable information that may be applied directly to breeding.

Maize is an important source of animal feed, and it is also a main staple crop that accounts for 15 to 56% of the total daily calories of humans in the developing countries (Food and Agriculture Organization, 2008). Tocopherol levels in maize kernels vary widely, however, αT only constitutes less than 20% of total tocopherols in maize kernels [19]. This wide range of variation and the relatively lower level provide a great opportunity to improve αT through genetic manipulation. Although previous linkage studies have detected multiple QTLs for αT, γT and δT [20], [21], the causative genes and polymorphisms have not yet been identified, presenting a challenge for genetic improvement. Recently, the availability of a maize reference genome sequence [22], the release of the first generation haplotype map of maize [23], and the development of high-throughput single nucleotide polymorphism (SNP) assays [24] enable genome-wide association studies (GWAS) to explore the genetic basis of tocopherol content. Such studies on natural allelic diversity should be complementary to mutant and transgenic studies conducted on tocopherol biosynthetic pathway genes in model organisms.

Thus, in this study, we used genome-wide association mapping with high-resolution SNP density in highly diverse maize germplasm to identify natural allelic variations that contribute to tocopherol levels in maize kernels. Three polymorphisms with independent effects were identified. The identification of these polymorphisms can not only further our understanding of mechanisms controlling natural tocopherol variation, but also provide markers for high vitamin E maize breeding.

## Results

### Genome-wide Association Analysis Identified One 2.4-Mb Genomic Region Significant for αT Content in Maize Kernels

An association panel comprising of 543 inbred lines [25] that represents global maize genetic diversity was genotyped using the Illumina MaizeSNP50 BeadChip containing 56,110 SNPs. These lines were subsequently processed through a quality control protocol (see Materials and Methods), and only 513 lines with high-quality genotypic data were retained for association analysis. These inbred lines showed considerable phenotypic variations for αT, γT, δT and total tocopherol content (TT, which is the sum of αT, γT and δT), with broad-sense heritabilities ranging from 0.50 for δT to >0.90 for γT, αT and TT (Table S1). To identify the genetic factors that control tocopherol levels in maize kernels, we conducted GWAS using a mixed linear model [26], [27] that accounts for population structure and individual relatedness (Figure S1).

**Figure 2:**
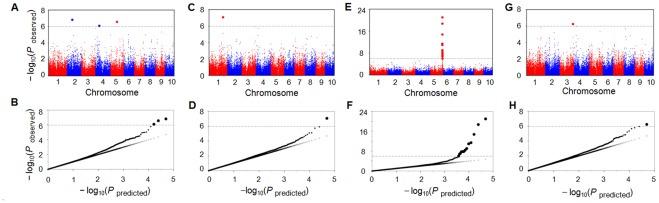
Manhattan plots and quantile-quantile plots of δ-tocopherol (A, B), γ-tocopherol (C, D), α-tocopherol (E, F) and total tocopherol (G, H). These plots are based on the association results in 513 lines using 48,962 SNPs. Each dot represents a SNP. The dashed line represents the Bonferroni-corrected significance level (∼0.05/48,962); SNPs that met this level were enlarged.

**Figure 3:**
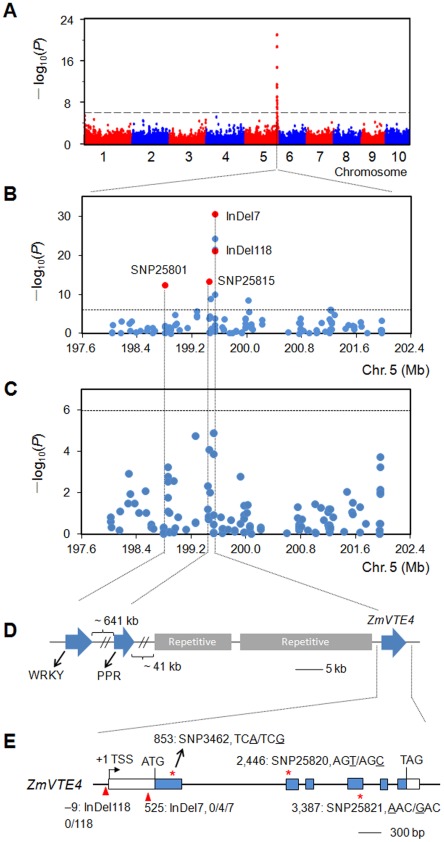
Association mapping results and genomic locations of significant polymorphisms for αT. (A) Association result for αT in a panel of 513 lines using 48,962 SNPs, showing the physical map locations of the SNPs (x-axis), the –log base 10 *P* values from a mixed linear model (y-axis), and the Bonferroni-adjusted significance threshold (dashed horizontal line). The spike at the end of chromosome 5 was located around *ZmVTE4*. (B) and (C) Regional plots showing association mapping results for SNPs located around *ZmVTE4* on chromosome 5 before (B) and after (C) controlling for the effects of InDel7, InDel118, SNP25801 and SNP25815 (red dots) in CAM478. The dashed lines represent the Bonferroni-adjusted significance threshold. (D) Genome organization upstream of *ZmVTE4*, showing repetitive sequences (grey boxes), the pentatricopeptide repeat (PPR) gene, and the WRKY transcription factor (WRKY). (E) Gene structure of *ZmVTE4* (exons, blue boxes; untranslated regions, open boxes) and polymorphisms’ locations (InDels, red triangles; SNPs, red asterisks). The location of the transcription start site was viewed as +1, and the location of the other polymorphisms were based on their relative distance from the transcription start site. The SNPs are given in the context of codon, with the SNPs underlined. InDel118 had two alleles: 0-bp (0) and 118-bp (118) insertions. InDel7 had three alleles: 0-bp (0), 4-bp (4), and 7-bp (7) insertions.

In total, twenty-four, five, three and six SNPs were identified to be significant for αT, δT, γT and TT, respectively, at a false discovery rate of 0.05 (Figure S2, Table S2). If a more stringent Bonferroni-corrected *P* value (1.02×10^−6^) was used as cutoff, thirteen, three, one and one SNPs were still significantly associated with αT, δT, γT and TT, respectively (Figure 2, Table S3). Based on the positive correlation between oil content and tocopherol levels (Table S4) [28], [29], a second association study excluding 35 high-oil lines from the original 513 lines was conducted in 478 lines (a population hereafter referred to as CAM478). This resulted in the re-identification of only nine of the same significant SNPs for αT (Figure 3B). However, none of the SNPs significantly associated with levels of δT, γT and TT in the panel of 513 lines were found significant in CAM478 (Table S3). To minimize the possibility of working on putative false associations, we only focused on the nine significant SNPs for αT that were detected in both the 513 and 478 lines.

The nine SNPs were located within a 2.4-Mb segment on chromosome 5 (198,800,752–201,222,043 bp, Figure 3B). Three of them, SNP3462, SNP25820 and SNP25821, were within *Zea mays VTE4* (*ZmVTE4*, Figure 3E), a gene that encodes γ-tocopherol methyltransferase which is involved in the rate-limiting conversion of γT to αT (Figure 1) [30]. The other SNPs were within other genes with no reported function on tocopherol levels. Because of the known function of *ZmVTE4* within tocopherol biosynthetic pathway (Figure 1), we first studied the three SNPs within *ZmVTE4*, and analyzed the effects of the other six SNPs while controlling for the associated effects of *ZmVTE4* SNPs.

### Identification of Putative Causative and Independent Polymorphisms within the 2.4-Mb Region for Kernel αT Content

Although the three *ZmVTE4* SNPs showed highly significant associations with αT in CAM478 (Figure 3B, Table S3), none of them were polymorphic in a By804/B73 recombinant inbred line (RIL) population, in which we identified a major QTL for αT near *ZmVTE4* (Figure S3) [21]. This indicates that there are other unknown causative variants within or linked to *ZmVTE4* in By804/B73 RILs. To identify these variants in a cost-effective manner, we re-sequenced full-length of *ZmVTE4* along with its 2,223-bp upstream and 282-bp downstream region in a subset of the whole association panel. These lines had previously been advocated for tocopherol association studies [31]. Robust associations of two InDels, InDel7 and InDel118, with the level of αT were detected (Table S5).

InDel7 was located within the 5**′** untranslated region (UTR) of *ZmVTE4*, and it had two more prevalent alleles in CAM478: a 0-bp insertion (allele0) and a 7-bp insertion (allele7), whereas a rare allele (allele4) having a 4-bp insertion was also present in only four high-oil lines (Figure 3E). InDel118 was located within the promoter region, 9-bp upstream of the putative transcriptional start site, and it had two alleles: a 0-bp insertion (allele0) and a 118-bp insertion (allele118) (Figure 3E). The level of linkage disequilibrium (LD) between these two InDels was low (*r* ^2^ = 0.15, Figure S4), and only InDel7 segregated in the By804/B73 (allele4/allele0) RIL population. This suggests InDel7 as a candidate causative polymorphism for the identified QTL.

InDel7 and InDel118 were genotyped in CAM478 with PCR-based markers (Figure S5). In a combined phenotypic data set across three environments, both InDels were significantly associated with αT (*P* = 2.7×10^−31^ and 8.6×10^−22^ for InDel7 and InDel118, respectively) (Table 1). Additionally, these associations were stable across environments (Table S6), suggesting that breeding with these markers would be effective in different environments. InDel7 and InDel118 were also weakly associated with γT (*P* = 1.2×10^−3^ and 1.3×10^−4^, respectively) and strongly associated with the ratio of αT to γT (denoted αT/γT) (*P* = 2.1×10^−14^ and 4.3×10^−23^, respectively), but no association was observed with TT (*P* = 4.0×10^−1^ and 6.9×10^−1^, respectively) (Table 1). This pattern of significant associations is consistent with the biochemical function of the enzyme encoded by *ZmVTE4*, that is, converting γT to αT.

**Table 1:** Summary of significant polymorphisms from genome-wide and candidate gene-based association studies.

Locus	Allele^a^	Frequency	*P* value (αT)	*P* value (γT)	*P* value (TT)	*P* value (αT/γT)	*R^2^* (αT, %)
InDel7	0/7	315/129	2.69×10^−31^	1.20×10^−3^	4.01×10^−1^	2.10×10^−14^	26.8
InDel118	0/118	131/321	8.63×10^−22^	1.34×10^−4^	6.91×10^−1^	4.30×10^−23^	19.3
SNP25801^b^	A/G	409/55	4.50×10^−13^	2.01×10^−2^	9.66×10^−1^	5.17×10^−9^	11.4
SNP25815^b^	T/C	238/206	4.83×10^−14^	9.00×10^−3^	8.75×10^−1^	8.90×10^−11^	12.4

a The favorable allele for αT is underlined. Alleles 7 and 0 indicate a 7-bp and 0-bp insertion at the InDel7 locus, respectively. Alleles 118 and 0 indicate a 118-bp and 0-bp insertion at the InDel118 locus, respectively. For InDel7, a third allele (allele4, 4-bp insertion) was only present in four high-oil lines.

b The SNP code from Illumina MaizeSNP50 BeadChip, the corresponding name and the source sequences of the SNP can be obtained from the Illumina website (Illumina). Both the *P* values and the phenotypic variations explained (*R^2^*) were from a mixed linear model controlling for population structure and individual relatedness. αT, α-tocopherol; γT, γ-tocopherol; TT, total tocopherol.

The association analysis in CAM478 identified six additional significant SNPs for αT on chromosome 5 that were not within *ZmVTE4* but co-located within the 2.4-Mb region (SNP25801, SNP25815, SNP25817, SNP53345, SNP51039 and SNP51045) (Figure 3, Table S3). All these SNPs were in weak LD with the three SNPs (SNP3462, SNP25820 and SNP25821) and two InDels (InDel7 and InDel118) from *ZmVTE4* (Figure S4). To investigate if these six SNPs remained significantly associated with αT after controlling for the effects of the *ZmVTE4* polymorphisms in CAM478, we fitted a mixed model with the three common haplotypes of InDel7-InDel118 as a covariate at each of these SNPs. Of the six SNPs, only SNP25801 and SNP25815 remained significantly associated with αT (*P* = 3.1×10^−13^ and 3.1×10^−10^, respectively). When we included InDel7, InDel118, SNP25801 and SNP25815 as covariates, no significant associations for other SNPs were detected (Figure 3B and 3C). SNP25801 was within the intron a gene encoding a WRKY transcriptional factor, while SNP25815 is located within the intron of a gene encoding a chloroplast targeting pentatricopeptide repeat (PPR) protein (Figure 3D).

### Confirmation of the Independent Contributions of Two InDels and one SNP to Kernel αT Content

**Figure 4:**
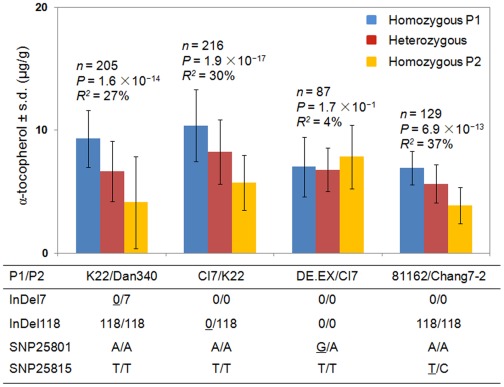
Associations between InDel7, InDel118, SNP25801, SNP25815 and αT content in four F_2∶3_ linkage populations. In each population (x-axis), the F_2_ individuals were divided into three groups based on observed genotypes (homozygous P1, homozygous P2 and heterozygous) of either of the four polymorphisms. The y-axis shows the average α-tocopherol content for each group within each population. The table shows which of the four polymorphisms segregated in each population. The favorable allele from association analysis is underlined. s.d., standard deviation.

To test the independence of InDel7, InDel118, SNP25801 and SNP25815, we developed four F_2∶3_ linkage populations that segregated at only one of those four polymorphisms (Figure 4, Table S7). Linkage population K22/Dan340 was polymorphic for InDel7 and monomorphic for all the other three polymorphisms, while CI7/K22, DE.EX/CI7 and 81162/Chang7-2 was polymorphic for InDel118, SNP25801 and SNP25815, respectively (Figure 4). The independent effect of SNP25815 for αT was confirmed in the 81162/Chang7-2 linkage population (*n* = 129, *P* = 6.9×10^−13^, Figure 4). Individuals with two T alleles (homozygous P1) showed significantly higher αT content than individuals with two C alleles (homozygous P2), in accordance to the results from the association panel. SNP25815 could explain 37% of αT variation in this population. Similar results were also observed for InDel7 and InDel118. However, no effect of SNP25801 was established (*n* = 87, *P* = 1.7×10^−1^, Figure 4). These results indicated that the effect of InDel7, InDel118 and SNP25815 is independent of each other, while SNP25801 might represent an association that is in LD with *ZmVTE4* or there are other unknown significant loci counteracting the effect of SNP25801 in the DE.EX/CI7 population.

Both InDel7 and InDel118 being located within *ZmVTE4*, we estimated the contribution of the InDel7-InDel118 haplotype to αT, γT and αT/γT variation in CAM478. The three common haplotypes accounted for 33% (αT), 5% (γT) and 26% (αT/γT) of the phenotypic variation (Table 2). The favorable haplotype (Allele0-Allele0) had 3.2-fold more αT content than the unfavorable haplotype (Allele7-Allele118). More interestingly, the favorable haplotype was present in about one third of total germplasm in the association panel (Table 2). This result suggested that germplasm adapted to local environments could be easily identified, thus facilitating the practical implementation of these two polymorphisms into maize breeding programs.

**Table 2:** Haplotype effects of InDel7 and InDel118 from *ZmVTE4.*

				Mean ± s.d.		
Haplotype	InDel7^a^	InDel118^b^	Frequency in GWAS panel	αT (µg/g)	γT (µg/g)	αT/γT
I	0	118	179	6.94 ± 3.12	27.18 ± 11.39	0.36 ± 0.30
II	0	0	118	10.43 ± 5.75	21.41 ± 12.95	0.83 ± 0.79
III	7	118	119	3.26 ± 2.82	28.97 ± 13.26	0.19 ± 0.19
IV	7	0	1	1.59	42.05	0.14
			*R^2^* (%)	33.2	4.9	25.6
			*P* value	4.8×10^−37^	3.3×10^−5^	3.5×10^−27^
			Fold change^c^	3.20	0.74	4.37

a The favorable allele for αT is underlined. Alleles 7 and 0 indicate a 7-bp and 0-bp insertion at the InDel7 locus, respectively.

b The favorable allele for αT is underlined. Alleles 118 and 0 indicate a 118-bp and 0-bp insertion at the InDel118 locus, respectively.

c Fold change was calculated between the most favorable haplotype (haplotype 0/0 because it had the highest amount of αT, which had the highest vitamin E activity) and the least favorable haplotype (haplotype 7/118). The *P* values and phenotypic variation explained (*R^2^*) were from a mixed linear model controlling for population structure and individual relatedness. Only the three common haplotypes (0/118, 0/0 and 7/118) were used to calculate *P* and *R^2^*. αT, α-tocopherol; γT, γ-tocopherol; TT, total tocopherol; s.d., standard deviation.

### InDel118 Likely Affected Kernel αT Content by Regulating Gene Expression

**Figure 5:**
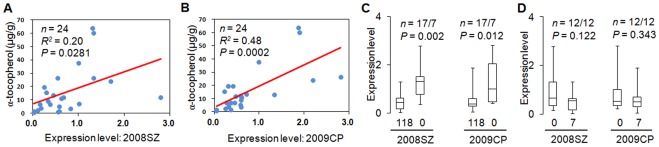
Expression analysis of *ZmVTE4*. (A) and (B) Plots of the results from correlation analyses between α-tocopherol content (y-axis) and *ZmVTE4* expression level in embryos collected at 20 days after pollination (x-axis) in two environments: Shangzhuang 2008 (2008SZ) and Changping 2009 (2009CP). (C) Box plot of *ZmVTE4* expression levels at each observed InDel118 allele across the two environments. The number before and after the slash are the number of lines for allele118 (118-bp insertion) and allele0 (0-bp insertion), respectively. (D) Box plot of *ZmVTE4* expression levels at each observed InDel7 allele across the two environments. The numbers before and after the slash are the number of lines for allele0 (0-bp insertion) and allele7 (7-bp insertion), respectively. For (C) and (D), the maximum, 75% quartile, median, 25% quartile and minimum expression levels are shown. The *P* values are based on two-sided Student’s *t-*tests.

The location of InDel7 and InDel118 in 5**′** UTR and promoter region, respectively, suggested that they may affect *ZmVTE4* expression. Therefore, we quantified the relative expression level of *ZmVTE4* in various inbred lines with varying levels of kernel αT (Table S8). *ZmVTE4* mRNA levels in embryos 20 days after pollination were positively correlated with αT content in both the 2008SZ (*n* = 24, *R^2^* = 0.20, *P* = 0.0281, Figure 5A) and 2009CP (*n* = 24, *R^2^* = 0.48, *P* = 0.0002, Figure 5B) experiments. In both environments, *ZmVTE4* expression level was significantly higher in the seven lines without the 118-bp insertion than in the other 17 lines with the 118-bp insertion (*P* = 0.002 and 0.012 in 2008SZ and 2009CP, respectively, Figure 5C). These results make biological sense because InDel118 is located 9-bp upstream of the putative transcription start site (Figure 3E), and thus, any insertion could change the distance between the TATA box and the transcription start site, leading to inefficient transcription initiation. In contrast, *ZmVTE4* expression levels and InDel7 were not correlated (*P* = 0.12 and 0.34 in 2008SZ and 2009CP, respectively, Figure 5D), suggesting that this polymorphism may perform its function through other regulating mechanism. Furthermore, although *ZmVTE4* expression levels in the seedling leaf, seedling root and endosperm vary in the same materials used for expression analysis in embryo, these variations were not associated with kernel αT content, and InDel118 did not affect *ZmVTE4* expression in these tissues either (Figure S6). These results suggested that enhancing *ZmVTE4* expression levels in other tissues might not lead to increased αT levels in maize kernels.

## Discussion

### Possible Regulatory Mechanisms Underlying Natural Kernel αT Content Variation

In this study, we performed GWAS on ∼500 diverse maize inbred lines analyzed in conjunction with ∼50,000 genome-wide SNPs, and identified a region on chromosome 5 showing robust association with αT content in the maize kernel. This region was also strongly associated with αT/γT and, to a lesser extent with γT. One gene, *ZmVTE4*, involved in tocopherol biosynthetic pathway was located within this region. Besides this region, some other SNPs were also identified to be significant for either tocopherol components or total tocopherol, however, no candidate genes from the biosynthetic pathway were identified near those SNPs (Table S2). Besides, not all the QTL detected in previous studies were identified in this study [20], [21]. Failure to detect those QTL have several possible reasons: some QTL may not be real, some QTL may involve low frequency polymorphisms which are hard to be detected by an association study, and the marker coverage in this study is still too low to meet the requirement of millions of markers for GWAS in maize diverse varieties [32].

Further sequencing of this chromosome 5 region led to the identification of two *ZmVTE4* InDels (InDel7 and InDel118) and one SNP (SNP25815) that are causative for the associations within this region. Linkage populations segregating at only one of these polymorphisms also confirmed their independent effect on αT (Figure 4). Those three polymorphisms might represent three different ways of regulation. Expression analysis showed that InDel118 affected *ZmVTE4* transcript level. Thus, InDel118 may control αT content through transcription regulation (Figure 5). Because *VTE4* is a well conserved gene from bacteria to gymnosperm to angiosperm [1], [16], we wonder whether the insertion is also present in other organisms. A search with the 118-bp sequences against the database didn’t identify any similar sequences in other species. We also analyzed some teosinte lines, both the 118-bp insertion and the 0-bp insertion were found. Those suggested that the insertion has appeared after the differentiation of maize from other species, but before the domestication of maize.

In contrast to InDel118, InDel7 had no effect on *ZmVTE4* mRNA level. However, a secondary RNA structure, a 7-bp and 11-bp motif separated by 51 nucleotides, was identified immediately upstream of InDel7 (Figure S7), and is similar to a structure involved in the translation efficiency regulation of chloroplast mRNA [33]. Although *ZmVTE4* is a nuclear-encoded gene, it is possible that the RNA structure of *ZmVTE4* has a similar function as that in chloroplast genes.

The identification of SNP25815 with αT suggested a third possible way of regulation. Although this SNP was within a PPR gene, the fact that this SNP is located only ∼85 kb upstream of *ZmVTE4* and the function of PPR gene on tocopherol had never been shown, lend support to the hypothesis that the association of SNP25815 with αT might represent its LD with an upstream *cis*-regulating element of *ZmVTE4*. In fact, long-distance upstream regulatory elements have been commonly found in maize, like what was seen with the *tb1* and *Vgt1* genes [34], [35]. Further deep sequencing of this region might shed more light on the real nature of the association of SNP25815 with αT.

The identification of InDel7, InDel118 and SNP25815 within a short region (<100 kb) adds evidence to the influence of allelic series on natural phenotypic variation, as what had been observed for flowering time and carotenoid content [36]–[38]. This allelic series had important implication for cloning of other QTLs. It indicated that more than one polymorphism might be underlying a single QTL even when the QTL was delineated to a less than 100-kb region. Also, this allelic series, plus the presence of other regulating genes far away or on other chromosomes, might be part of the reason why maize had so wide range of phenotypic variation. Thus, identifying such allelic series would be an important way towards understanding the genetic architecture of quantitative traits.

### Different Contributions of Tocopherol Biosynthetic Pathway Genes to Natural Phenotypic Variation

Candidate gene based association studies have been widely used to determine the genetic basis of traits with defined pathway in maize, such as flowering time, starch content and carotenoid content (reviewed in [39]). While there are usually numerous genes in a specific pathway, only a few genes have been studied or found to be associated with natural phenotypic variation. It is not clear whether the insignificant genes have limited function on natural phenotypic variation, or the functional polymorphisms, such as long-distance upstream regulating elements, were not being identified. With the Illumina SNP platform, for the first time, we can look at this question for tocopherol content in maize kernels for all the pathway genes (Figure 1) and their upstream regulating elements. Based on our current results, polymorphisms within *ZmVTE4* contributed to a large proportion of natural tocopherol variation (Table 2), however, none of the SNPs from the other five tocopherol biosynthesis pathway genes (*ZmHPPD*, *ZmVTE1*, *ZmVTE2*, *ZmVTE3*, *ZmVTE5*, Figure 1) were associated with the variation in tocopherol levels. Although the coverage of this platform is not high enough, we should still be able to identify significant SNPs around these genes if the effect size of the functional polymorphisms in these genes are large and are not rare, as what was seen in the *ZmVTE4* region (Figure S4). Additionally, our expression analysis showed no correlation between the expression levels of these genes and tocopherol levels (Table S9). Thus, our failure to detect significant associations within these five biosynthetic pathway genes favored the possibility that these genes might have limited contribution to natural tocopherol variations. This possibility is also in agreement with previous QTL studies where not all the candidate genes were within identified QTL regions [20], [21]. These findings suggested a limited power of candidate gene-based association studies in identifying genetic variations underlying phenotypic diversity, even for a trait that has a well characterized biosynthetic pathway. Thus, a larger set of SNPs or other polymorphisms are needed to capture the genetic diversity present in maize genome, which can then help to identify new variants within or beyond the genes from the metabolic pathway.

### Potential Application of the Identified Polymorphisms for Marker-Assisted Selection

Although vitamin E has health benefits for human and animals, dietary intake is usually not met. Natural tocopherols are the superior source of vitamin E compared with synthetic tocopherols [40], therefore, enhancing αT content in important crops would be a feasible and efficient way to improve vitamin E nutrition in both humans and animals. Although lots of previous studies confirmed the biochemical function of *ZmVTE4* on αT through mutant or transgenic studies [1], [16], none of them identified naturally existing polymorphisms that can be used for marker-assisted selection. Our results validated the large effects of InDel7 and InDel118 on αT content in maize kernels (Table 2). Based on the high frequency of the favorable alleles in the germplasm used in this study, it is likely that the favorable alleles are already present in locally-adapted maize germplasm throughout the world, which could be used to enhance αT levels in a wide range of genetic backgrounds. There is no current data to support the actual breeding value of InDel7 and InDel118, however, in view of the genetic effect of InDel7 and InDel118, the projected breeding value of these two InDels has important practical consequences. The average αT content in maize kernels of CAM478 is 6.7 µg/g, which would require 2.2 kg of maize kernels to meet the 15 mg dietary reference intake (DRI) of αT per day [41]. However, there is a 3.2-fold difference between the best and the worst haplotypes of InDel7 and InDel118 (Table 2), a marker-assisted selection program that selects on the favorable alleles of these two polymorphisms could increase the average level of αT content to 21.4 µg/g, thus, only ∼0.7 kg of maize kernels would be required to attain the DRI. Although 0.7 kg is still a large amount, combination of these markers identified here with the other yet-to-be-identified polymorphisms would further reduce the amount of needed maize to meet the DRI.

## Materials and Methods

### Association Panel, Genotyping, Quality Control and Population Structure

The association panel consisted of 543 diverse lines, including 242 lines from the International Maize and Wheat Improvement Center (CIMMYT), 234 lines from China and 67 lines from the USA. Most of the CIMMYT lines were of tropical or subtropical origin, whereas most lines from the USA and China were of temperate origin. Detailed information on 525 of these lines can be found in a previous study [25]. All the lines were genotyped using the Illumina MaizeSNP50 BeadChip (Illumina), which consists of 56,110 SNPs. The quality of each SNP was checked manually as previously reported [42], and SNPs with bad quality were recorded and excluded from further analysis.

All maize lines used in the association panel were inbred, and hence should be homozygous for most of the SNPs. Because the two homozygous states, namely AA and BB, were randomly labeled with fluorescence, an AA to BB ratio of approximately one would be expected. However, 21 lines had a ratio of 2.42–136.91. Most of these lines were located near each other on the chips, and principal component analysis showed that they formed a separate cluster; therefore, they were excluded from further analysis. We also excluded nine lines with a high missing genotyping rate (>50%) or a high heterozygous rate (>33%). Therefore, only 513 lines with high-quality genotypic data were used for the final association analysis.

To estimate population structure and kinship coefficients [26], the missing rate (M), heterozygous rate (H) and minor allele frequency (MAF) were calculated for all SNPs in the 513 lines. SNPs with M >20%, H >20% or MAF <10% were excluded. Additionally, SNPs with multiple or unknown chromosomal locations were also excluded. After this filtering, 36,618 SNPs were used to estimate Q using STRUCTURE [43], [44]. Using 1,536 SNPs, this panel was previously divided into three subgroups [25], and hence, we calculated Q assuming three subgroups. The kinship matrix was calculated with the same 36,618 SNPs using the method of Loiselle et al. [45].

### *ZmVTE4* Re-sequencing and Analysis

The sequence of maize γ-tocopherol methyltransferase (*ZmVTE4*) was retrieved from NCBI database (www.ncbi.nlm.nih.gov) by blasting *Arabidopsis* protein sequences against maize high-throughput genomic sequences. Primers were designed using Primer Premier 5 and Primer 3 (Table S10). Sequencing was performed using 3730 sequencers. The sequences were aligned using MUSCLE [46], and refined manually in BioEdit [47]. Genotyping of two *ZmVTE4* InDels (InDel7 and InDel118) in CAM478 was performed with PCR based markers (Tables S10 and S11). All the sequences can be accessed through NCBI under the accession number JQ246100-JQ246337.

### Field Experiments and Phenotyping

All 543 lines of the association panel were planted in 2009 at three locations, namely Sichuan, Yunnan and Hainan, China. These lines were divided into two groups, temperate and tropical/subtropical, based on germplasm adaptation. A subpanel of 155 lines [31] was previously planted in Beijing, China during 2006 and 2007. All the lines were planted in a single row plot with two replications using a complete randomized block design across all the locations. All lines were self-pollinated, and ears were air-dried before manual shelling. Kernels from the middle of at least three ears in each replicate were used for phenotypic analysis. Tocopherol extraction and detection was carried out as previously reported [21]. Levels of three tocopherol compounds, namely αT, γT and δT, were measured, and total tocopherol was calculated as the sum of αT, γT and δT.

### Linkage Analysis

Four crosses were made using 6 diverse maize inbred lines (Tables S7). The CI7/K22 and K22/Dan340 populations were planted in Beijing, China in 2009, whereas the 81162/Chang7-2 and DE.EX/CI7 populations were planted in Hainan, China in 2010. For each cross, thirteen F_2_ plants per row were planted, and the number of rows varies according to the total number of individuals in each cross. Individual plants were self-pollinated. Harvested ears were air-dried and shelled. Kernels from the middle of each ear were used for phenotyping. A one-way ANOVA model was fitted within each cross to assess the association between genotypes and tocopherol content. Each model had the compound content as the response variable and the polymorphism that segregated in the given cross as the factor.

### Expression Analysis

Embryos and endosperms from developing seeds were collected 20 days after pollination (Table S8). Root and leaf samples were collected from nine days old seedlings. Total RNA was prepared using the Bioteke RNA extraction kit (Bioteke). Recombinant Molony Murine Leukemia Virus Reverse Transcriptase and an oligo (dT) primer (Promega) was used to synthesize the complementary DNA. Real time quantitative RT-PCR was used to analyze the relative mRNA abundance in each line with the Ex Taq premix (Takara Shuzo) and the primers listed in Table S10. Three replicates of each primer/tissue combination were obtained. Relative expression levels of each gene were calculated using the 2^–ΔΔCT^ method [48] with actin as the endogenous control. Correlation analysis and two-sided Student’s *t*-tests were performed in SAS (SAS Institute Inc.).

### Statistical Analyses

The association panel was phenotyped in multi-environments, therefore, we fitted a model to obtain an unbiased estimation of each tocopherol compound in each line:

Y_ijk_ = µ+Environment_i_+Replicate (Environment)_ij_+Line_k_+(Environment × Line)_ik_+ε_ijk._where Y_ijk_ is the observed phenotype for the k^th^ line in the j^th^ replicate of the i^th^ environment, µ is the grand mean, Environment_i_ is the random effect of the i^th^ environment, Replicate (Environment)_ij_ is the random effect of the j^th^ replicate in the i^th^ environment, Line_k_ is the random effect of the k^th^ line, (Environment × Line)_ik_ is the random interaction effect of the i^th^ environment and the k^th^ line, and ε_ijk_ is the error term, which follows an independent, identically distributed N (0, σ^2^_e_) distribution.

The MIXED procedure in SAS (SAS Institute Inc.) was used to get the best linear unbiased estimate (BLUP) of the line effect, which was then added to the estimate of the grand mean. The resulting values were used as phenotypes for the association analysis. The GLM procedure in SAS (SAS Institute Inc.) was used to calculate the mean squares. These were used to estimate the heritability and 95% confidence interval of each tocopherol-related trait according to the method of Knapp et al. [49].

We used the “Q + K” model [26], [27] implicated in TASSEL [50] to perform association analysis. Only SNPs or InDels with MAF ≥0.05 and a missing rate <25% were used. A similar model was used to calculate the phenotypic variation explained by each polymorphism in TASSEL.

## Supporting Information

Figure S1 **Summary of population structure (A) and individual relatedness (B) in the association panel of 513 inbred lines.** (A) Three ancestral populations were detected (depicted in green, blue and red). Each vertical line represents an inbred line, and the membership percentages of each inbred line belonging to each of the three ancestral populations were depicted by the height of the colored vertical line. The three ancestral populations were re-named as: SS, stiff stalk lines; NSS, non-stiff stalk lines; TST, tropical or subtropical lines. (B) Histogram depicting the percentage distribution of pairwise kinship coefficients. A total of 131,328 (513×512/2, where 513 is the number of lines in the association panel) kinship coefficients were used. Only kinship coefficients <0.50 are shown; 0.24% of kinship coefficients were >0.50.
(TIF)

Figure S2 **Manhattan plots for δ-tocopherol (A), γ-tocopherol (B), α-tocopherol (C) and total tocopherol (D).** These plots are based on the association results in 513 lines using 48,962 SNPs. Raw *P* values were adjusted using false discovery rate and named q. Each dot represents a SNP. The dashed line represents threshold of significance level ( = -lg0.05); SNPs that met this level were enlarged.
(TIF)

Figure S3 **Linkage mapping results on chromosome 5 in a RIL population derived from By804 and B73.** The x-axis is the genetic map position, and the y-axis is the LOD score. The dashed grey line is the empirical significance threshold (LOD  = 2.5). A QTL for αT, γT and αT/γT was identified, and no significant QTL was mapped for total tocopherol. Also, *ZmVTE4* was mapped within the QTL interval for αT, γT and αT/γT. This result was based on previous results [21]. αT, α-tocopherol; γT, γ-tocopherol; TT, total tocopherol.
(TIF)

Figure S4 **Pairwise LD between 11 significant polymorphisms for αT that were within a 2.4-Mb interval on chromosome 5 including** ***ZmVTE4*** **.** These polymorphisms are shown according to their relative positions on chromosome 5. *r^2^* values were shown below the diagonal and *r^2^*>0.1 are highlighted in pink. *P* values are shown above the diagonal. Five of the 11 polymorphisms, InDel118, InDel7, SNP3462, SNP25820 and SNP25821, were from *ZmVTE4*.
(TIF)

Figure S5 **PCR assays for InDel7 (A) and InDel118 (B) of** ***ZmVTE4*** **.** For each polymorphism, the segregation of the PCR product (left panel) and a schematic diagram of the PCR amplicon (right panel) are shown. The primer sequences and detailed PCR reaction procedures are given in Tables S10 and S11.
(TIF)

Figure S6 ***ZmVTE4*** **expression analysis in the endosperm, leaf and root.** (A, B and C) Plots of the results from correlation analyses between α-tocopherol content and *ZmVTE4* expression level in the endosperm (A), leaf (B) and root (C). (D) Box plot of *ZmVTE4* expression levels at each observed InDel118 allele across the three tissues. The number before and after the slash are the number of lines for allele118 (118-bp insertion) and allele0 (0-bp insertion), respectively. The maximum, 75% quartile, median, 25% quartile and minimum expression levels are given. The *P* values are based on two-sided Student’s t-tests.
(TIF)

Figure S7 **RNA secondary structure in the 5′ UTR of** ***ZmVTE4*** **aligned to the standard motif identified by Schmitz-Linneweber et al.** [**33**] **.** The 7-bp motif and the 11-bp motif (blue boxes) were separated by 51 nucleotides (51 nt). InDel7 from *ZmVTE4* is in the red box. 0, 0-bp insertion; 4, 4-bp insertion; 7, 7-bp insertion; n, any of the four nucleotides.
(TIF)

Table S1 **Phenotypic variation, heritability and correlation analysis in the association panel containing 513 lines.** ^a^ANOVA, analysis of variance, showing the mean square and degrees of freedom (in parentheses). The F-test was applied to determine the significance level. Both the environments and lines were fitted in the model as random effects. ^b^95% confidence interval for broad-sense heritability. ^c^The number above and below the diagonal is the genetic and phenotypic correlation coefficients, respectively. **, *P*<0.01; s.d., standard deviation.
(DOCX)

Table S2 **Summary of association results based on false discovery rate.** ^a^The SNP code from Illumina MaizeSNP50 BeadChip. The corresponding name and source sequences of the SNPs can be obtained from the Illumina website (Illumina). SNPs with a false discovery rate less than 0.05 in the association panel of 513 lines were reported in this table. ^b^The favorable allele is underlined. ^c^Candidate genes were based on Figure 1, gene located within 5 Mb upstream or downstream of a SNP was considered to be a candidate gene for that SNP.
(DOCX)

Table S3 **Summary of association results before and after excluding high-oil lines.**
^a^The SNP code from Illumina MaizeSNP50 BeadChip. The corresponding name and source sequences of the SNPs can be obtained from the Illumina website (Illumina). SNPs with a significance level less than 1.02×10^−6^ in the association panel of 513 lines were reported in this table. ^b^The favorable allele is underlined. c SNPs that were significant at *P*<1.02×10^−6^ in CAM478 are in bold red font. NA, not available because the minor allele frequency was less than 0.05.
(DOCX)

Table S4 **Effect of oil content on tocopherol content.** These results are based on the linear unbiased estimate of the 513 lines across three locations. a Significance level from a one-way ANOVA model, where the given compound content was the response variable and high-oil lines versus normal lines was the factor. s.d., standard deviation.
(DOCX)

Table S5 **Association results for two polymorphisms from** ***ZmVTE4*** **with α-tocopherol in a subpanel of 155 lines.** ^a^The favorable allele is underlined. InDel7 had three alleles, 0/4/7, with allele4 present in only four lines, allele4 and allele7 were combined because both alleles were unfavorable. 0, 0-bp insertion; 4, 4-bp insertion; 7, 7-bp insertion; 118, 118-bp insertion. ^b^The best linear unbiased prediction (BLUP) of each line across the two environments (Beijing 2006 and Beijing 2007) was calculated using a model in which the compound content was the responsive variable and the line was a random effect (see details in Methods). This model was fitted using the MIXED procedure in SAS. ^c^The phenotypic variation for αT explained by each polymorphism in each environment was calculated using the “Q + K” model in TASSEL. ^d^Allele frequency in the whole subpanel. e Allele frequency in a subpanel excluding high-oil lines. n.d., not detected; n.s., not significant; αT, α-tocopherol; γT, γ-tocopherol.
(DOCX)

Table S6 **Association of four polymorphisms with tocopherol-related traits across three environments in CAM478.** The *P* values are from a “Q + K” model fitted in TASSEL. HN, Hainan; SC, Sichuan; YN, Yunnan; n.s., not significant.
(DOCX)

Table S7 **Evaluation of the genetic effect of four polymorphisms on tocopherol traits in F2∶3 populations.** ^a^ *n* is the number of F2 individuals. A one-way ANOVA model with the compound as the response variable and the segregating polymorphism as the factor was used to get the significance level and phenotypic variation explained (*R* ^2^). αT, α-tocopherol; γT, γ-tocopherol; TT, total tocopherol; ND, not detected; N.P., not polymorphic; n.s., not significant. The favorable allele is underlined in each population.
(DOCX)

Table S8 **Inbred lines used for expression analysis.** The phenotypic data for α-tocopherol is based on the field experiment with the 155 lines in 2006 and 2007. Presence (√) and absence (NA, not available) are indicated for *ZmVTE4* expression data. 2008SZ, Shangzhuang 2008; 2009CP, Changping 2009.
(DOCX)

Table S9 **Summary of correlation between expression levels of five genes from tocopherol biosynthesis pathway and tocopherol content.** The embryos collected at 20 days after pollination (*n* = 24) were used to perform quantitative RT-PCR of each gene. The tocopherol content was averaged over two years (Beijing 2006, 2007). The CORR procedure in SAS was used to obtain the Pearson correlation coefficients (r). “–” represents negative correlation.
(DOCX)

Table S10 **Primers used in the study.**
(DOCX)

Table S11 **PCR reaction system and protocol for InDel7 and InDel118 of** ***ZmVTE4*** **.**
(DOCX)
