# Supplementary figures and images for: Genome-Wide Association Studies Identified Three Independent Polymorphisms Associated with α-Tocopherol Content in Maize Kernels
Source: PLoS One. 2012 May 15;7(5):e36807. doi: 10.1371/journal.pone.0036807 (PMC3352922; doi:10.1371/journal.pone.0036807)

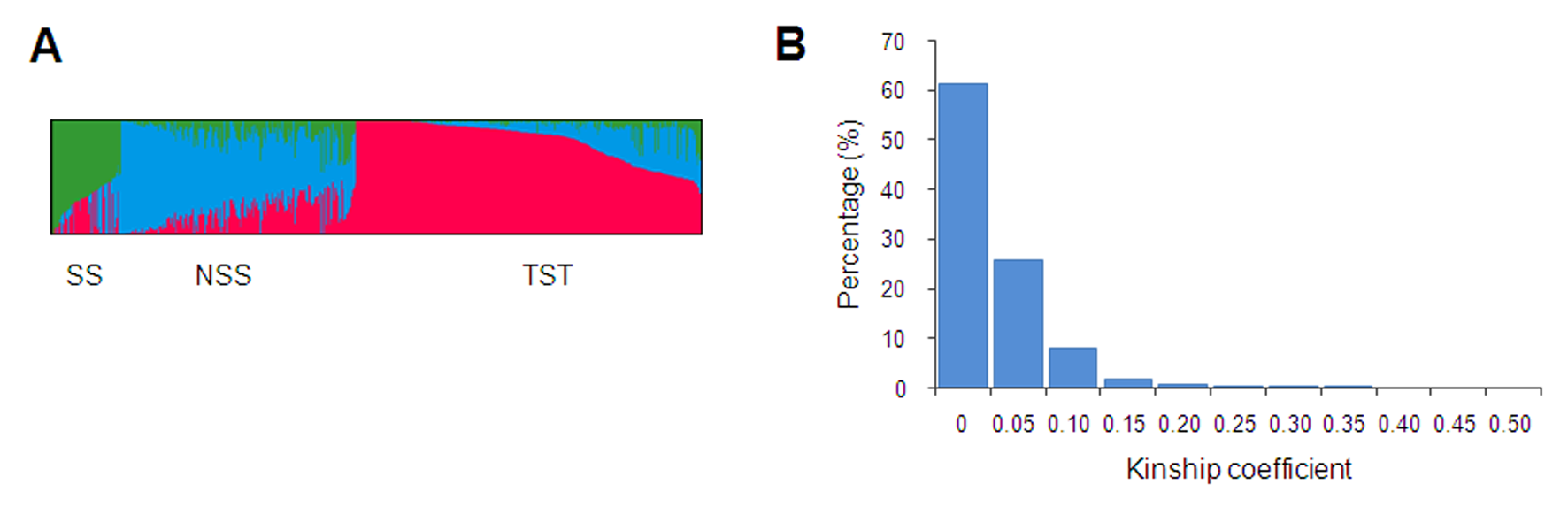

Supplement: Figure S1 — Summary of population structure (A) and individual relatedness (B) in the association panel of 513 inbred lines. (A) Three ancestral populations were detected (depicted in green, blue and red). Each vertical line represents an inbred line, and the membership percentages of each inbred line belonging to each of the three ancestral populations were depicted by the height of the colored vertical line. The three ancestral populations were re-named as: SS, stiff stalk lines; NSS, non-stiff stalk lines; TST, tropical or subtropical lines. (B) Histogram depicting the percentage distribution of pairwise kinship coefficients. A total of 131,328 (513×512/2, where 513 is the number of lines in the association panel) kinship coefficients were used. Only kinship coefficients <0.50 are shown; 0.24% of kinship coefficients were >0.50. (TIF) [file pone.0036807.s001.tif]

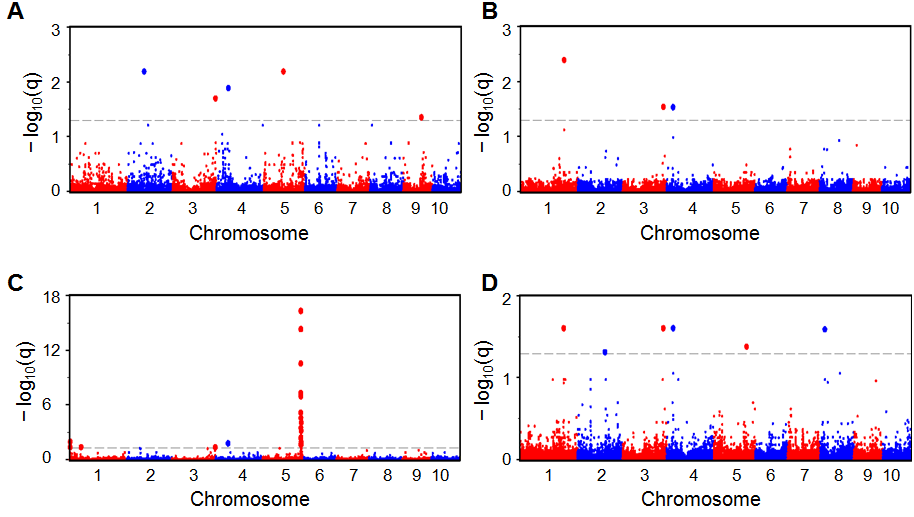

Supplement: Figure S2 — Manhattan plots for δ-tocopherol (A), γ-tocopherol (B), α-tocopherol (C) and total tocopherol (D). These plots are based on the association results in 513 lines using 48,962 SNPs. Raw P values were adjusted using false discovery rate and named q. Each dot represents a SNP. The dashed line represents threshold of significance level ( = -lg0.05); SNPs that met this level were enlarged. (TIF) [file pone.0036807.s002.tif]

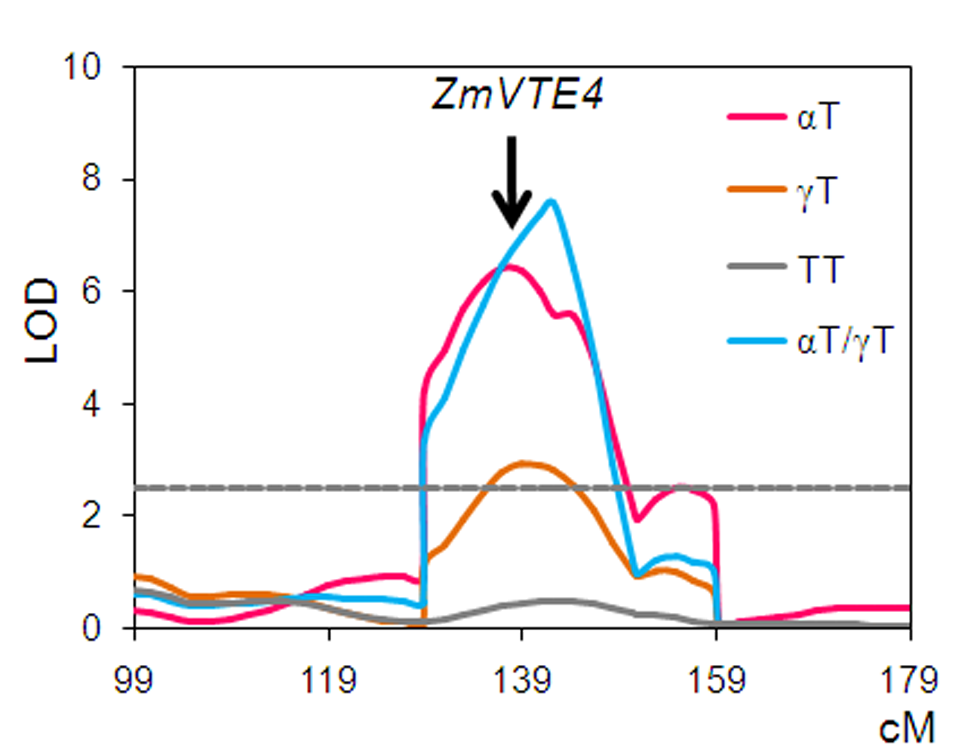

Supplement: Figure S3 — Linkage mapping results on chromosome 5 in a RIL population derived from By804 and B73. The x-axis is the genetic map position, and the y-axis is the LOD score. The dashed grey line is the empirical significance threshold (LOD = 2.5). A QTL for αT, γT and αT/γT was identified, and no significant QTL was mapped for total tocopherol. Also, ZmVTE4 was mapped within the QTL interval for αT, γT and αT/γT. This result was based on previous results [21]. αT, α-tocopherol; γT, γ-tocopherol; TT, total tocopherol. (TIF) [file pone.0036807.s003.tif]

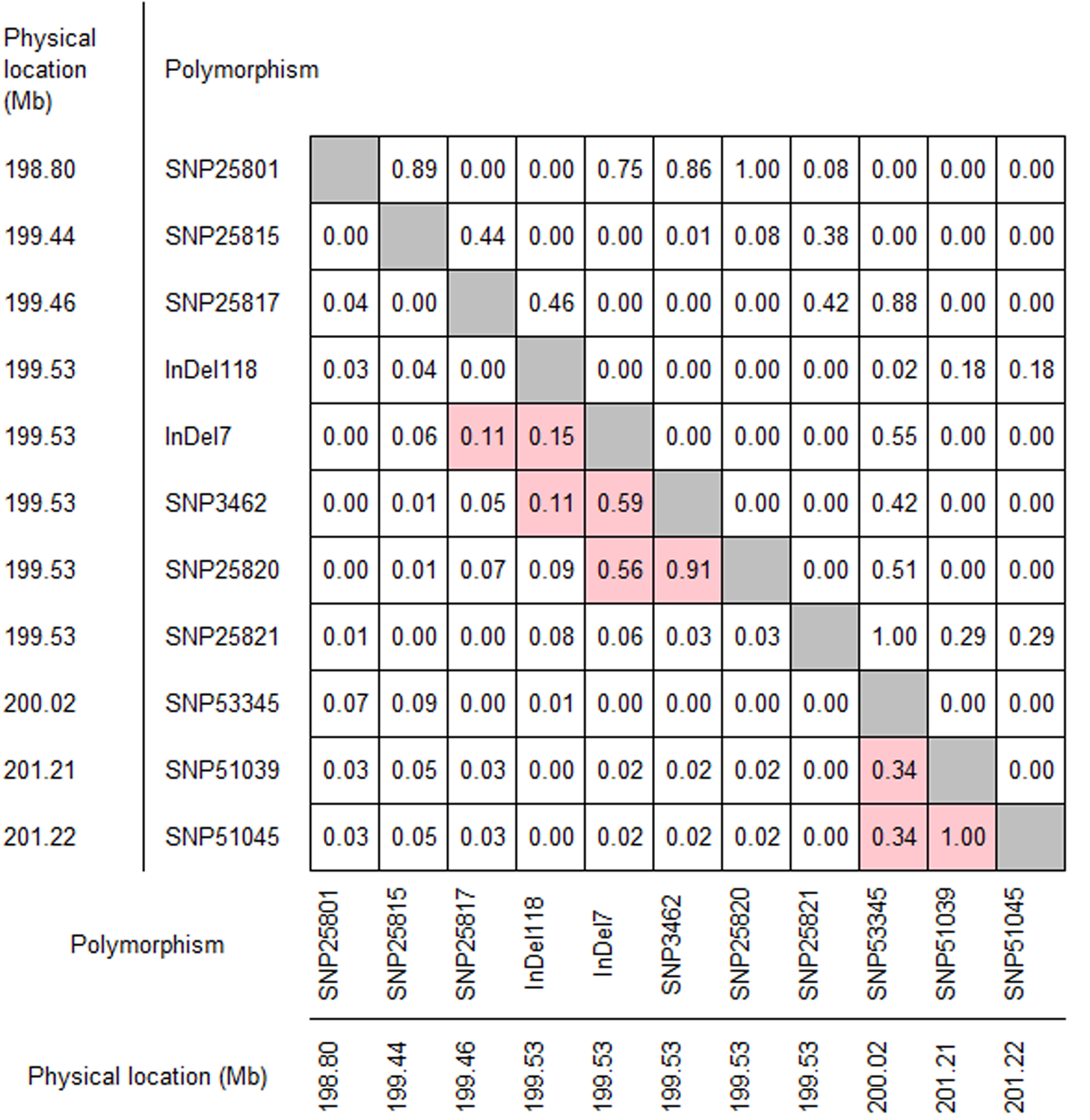

Supplement: Figure S4 — Pairwise LD between 11 significant polymorphisms for αT that were within a 2.4-Mb interval on chromosome 5 including ZmVTE4 . These polymorphisms are shown according to their relative positions on chromosome 5. r2 values were shown below the diagonal and r2>0.1 are highlighted in pink. P values are shown above the diagonal. Five of the 11 polymorphisms, InDel118, InDel7, SNP3462, SNP25820 and SNP25821, were from ZmVTE4. (TIF) [file pone.0036807.s004.tif]

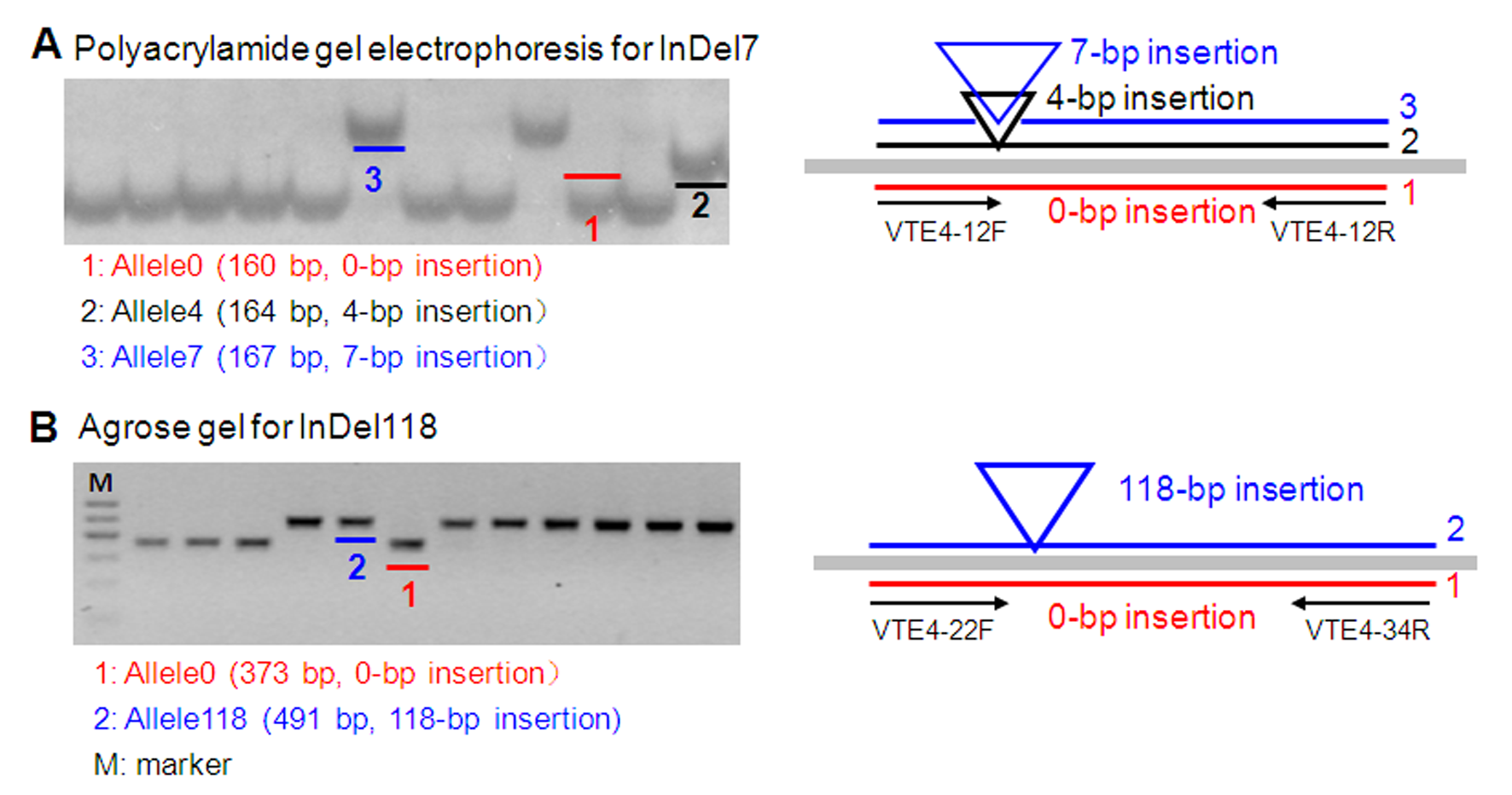

Supplement: Figure S5 — PCR assays for InDel7 (A) and InDel118 (B) of ZmVTE4 . For each polymorphism, the segregation of the PCR product (left panel) and a schematic diagram of the PCR amplicon (right panel) are shown. The primer sequences and detailed PCR reaction procedures are given in Tables S10 and S11. (TIF) [file pone.0036807.s005.tif]

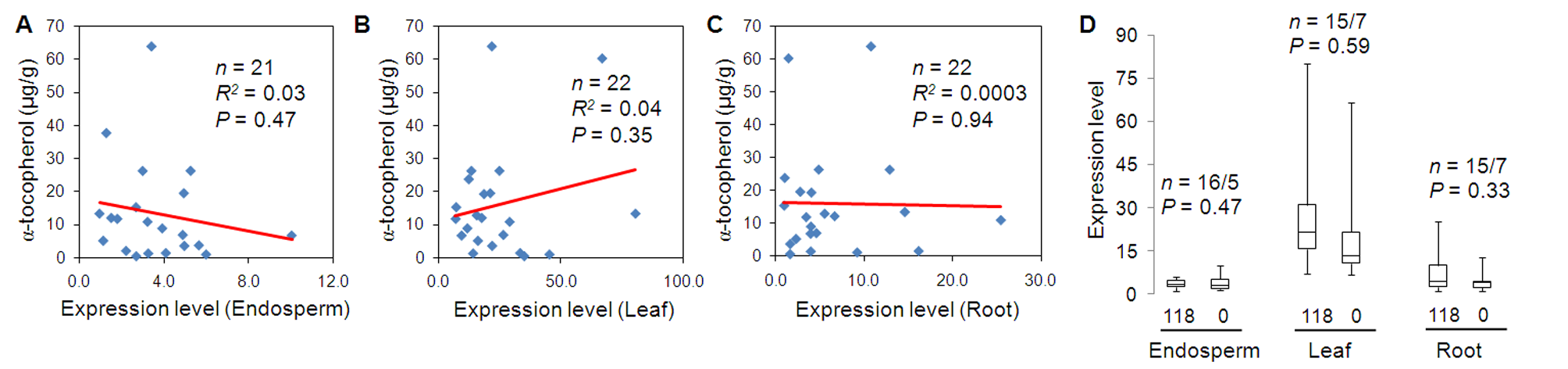

Supplement: Figure S6 — ZmVTE4 expression analysis in the endosperm, leaf and root. (A, B and C) Plots of the results from correlation analyses between α-tocopherol content and ZmVTE4 expression level in the endosperm (A), leaf (B) and root (C). (D) Box plot of ZmVTE4 expression levels at each observed InDel118 allele across the three tissues. The number before and after the slash are the number of lines for allele118 (118-bp insertion) and allele0 (0-bp insertion), respectively. The maximum, 75% quartile, median, 25% quartile and minimum expression levels are given. The P values are based on two-sided Student’s t-tests. (TIF) [file pone.0036807.s006.tif]

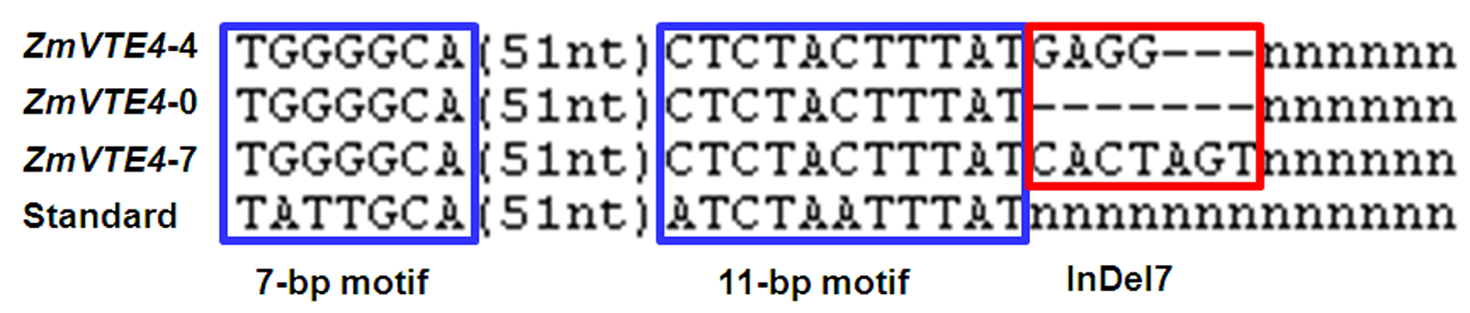

Supplement: Figure S7 — RNA secondary structure in the 5′ UTR of ZmVTE4 aligned to the standard motif identified by Schmitz-Linneweber et al. [33] . The 7-bp motif and the 11-bp motif (blue boxes) were separated by 51 nucleotides (51 nt). InDel7 from ZmVTE4 is in the red box. 0, 0-bp insertion; 4, 4-bp insertion; 7, 7-bp insertion; n, any of the four nucleotides. (TIF) [file pone.0036807.s007.tif]
