# Supplementary material for: Genome-Wide Association Studies Identified Three Independent Polymorphisms Associated with α-Tocopherol Content in Maize Kernels
Source: PLoS One. 2012 May 15;7(5):e36807. doi: 10.1371/journal.pone.0036807 (PMC3352922; doi:10.1371/journal.pone.0036807)
Supplement: Table S1 — Phenotypic variation, heritability and correlation analysis in the association panel containing 513 lines. aANOVA, analysis of variance, showing the mean square and degrees of freedom (in parentheses). The F-test was applied to determine the significance level. Both the environments and lines were fitted in the model as random effects. b95% confidence interval for broad-sense heritability. cThe number above and below the diagonal is the genetic and phenotypic correlation coefficients, respectively. **, P<0.01; s.d., standard deviation. (DOCX) [file pone.0036807.s008.docx]

**Table S1. Phenotypic variation, heritability and correlation analysis in the association panel containing 513 lines**

| Category | Source of variation | δ-tocopherol (μg/g) | γ-tocopherol (μg/g) | α-tocopherol (μg/g) | Total tocopherol (μg/g) |
| --- | --- | --- | --- | --- | --- |
| Descriptive | Range | 0.55–8.94 | 3.37–141.25 | 0.40–61.08 | 7.45–198.70 |
| statistics | Mean ± s.d. | 1.41 ± 0.74 | 30.07 ± 19.38 | 7.56 ± 6.55 | 38.15 ± 22.99 |
|  |  |  |  |  |  |
| ANOVA^a^ | Environments | 40.04 (2)** | 851.15 (2)** | 273.39 (2)** | 601.24 (2)** |
|  | Lines | 3.25 (323)** | 994.25 (502)** | 106.40 (500)** | 1376.84 (502)** |
|  | Error | 1.63 (241) | 59.78 (712) | 7.00 (671) | 78.74 (722) |
|  | Heritability | 0.50 | 0.94 | 0.93 | 0.94 |
|  | 95% CI for H^b^ | 0.36–0.60 | 0.93–0.95 | 0.92–0.94 | 0.93–0.95 |
|  |  |  |  |  |  |
| Correlation | δ-tocopherol | – | 0.36** | 0.14** | 0.38** |
| coefficients^c^ | γ-tocopherol | 0.40** | – | 0.31** | 0.96** |
|  | α-tocopherol | 0.12** | 0.29** | – | 0.56** |
|  | Total tocopherol | 0.42** | 0.96** | 0.52** | – |

^a^ ANOVA, analysis of variance, showing the mean square and degrees of freedom (in parentheses). The F-test was applied to determine the significance level. Both the environments and lines were fitted in the model as random effects. ^b^ 95% confidence interval for broad-sense heritability. ^c^ The number above and below the diagonal is the genetic and phenotypic correlation coefficients, respectively. **, *P* < 0.01; s.d., standard deviation.
