# Supplementary material for: Genome-Wide Association Studies Identified Three Independent Polymorphisms Associated with α-Tocopherol Content in Maize Kernels
Source: PLoS One. 2012 May 15;7(5):e36807. doi: 10.1371/journal.pone.0036807 (PMC3352922; doi:10.1371/journal.pone.0036807)
Supplement: Table S2 — Summary of association results based on false discovery rate. aThe SNP code from Illumina MaizeSNP50 BeadChip. The corresponding name and source sequences of the SNPs can be obtained from the Illumina website (Illumina). SNPs with a false discovery rate less than 0.05 in the association panel of 513 lines were reported in this table. bThe favorable allele is underlined. cCandidate genes were based on Figure 1, gene located within 5 Mb upstream or downstream of a SNP was considered to be a candidate gene for that SNP. (DOCX) [file pone.0036807.s009.docx]

**Table S2. Summary of association results based on false discovery rate**

| SNP^a^ | Chromosome | Position | Allele^b^ | Frequency in 513 lines | FDR in 513 lines | Candidate gene^c^ |
| --- | --- | --- | --- | --- | --- | --- |
| α-tocopherol | | | | | | |
| 41,597 | 1 | 3,009,997 | A/G | 99/401 | 4.16 × 10^-2^ |  |
| 54,850 | 1 | 3,379,175 | A/C | 315/177 | 1.05 × 10^-2^ |  |
| 1,213 | 1 | 60,212,244 | A/G | 472/31 | 4.03 × 10^-2^ |  |
| 17,550 | 3 | 228,955,373 | A/T | 424/49 | 4.16 × 10^-2^ |  |
| 18,772 | 4 | 65,286,187 | C/G | 433/61 | 1.55 × 10^-2^ |  |
| 18,773 | 4 | 65,286,238 | A/G | 62/436 | 1.55 × 10^-2^ |  |
| 25,801 | 5 | 198,800,752 | A/G | 439/60 | 4.31 × 10^-8^ | *ZmVTE4* |
| 25,814 | 5 | 199,266,329 | A/G | 331/160 | 2.02 × 10^-2^ | *ZmVTE4* |
| 25,815 | 5 | 199,442,506 | T/C | 257/221 | 2.56 × 10^-11^ | *ZmVTE4* |
| 49,239 | 5 | 199,444,812 | A/G | 344/140 | 4.18 × 10^-3^ | *ZmVTE4* |
| 25,817 | 5 | 199,461,718 | A/G | 355/125 | 1.05 × 10^-7^ | *ZmVTE4* |
| 3,462 | 5 | 199,528,414 | A/G | 99/399 | 4.25 × 10^-17^ | *ZmVTE4* |
| 25,820 | 5 | 199,530,006 | T/C | 95/397 | 4.25 × 10^-15^ | *ZmVTE4* |
| 25,821 | 5 | 199,530,947 | A/G | 266/207 | 6.72 × 10^-6^ | *ZmVTE4* |
| 408 | 5 | 199,970,863 | A/G | 216/263 | 3.53 × 10^-3^ | *ZmVTE4* |
| 53,345 | 5 | 200,017,666 | T/C | 418/71 | 2.6 × 10^-5^ | *ZmVTE4* |
| 25,826 | 5 | 200,023,434 | T/G | 232/248 | 3.12 × 10^-4^ | *ZmVTE4* |
| 45,458 | 5 | 200,776,518 | A/G | 46/452 | 1.05 × 10^-2^ | *ZmVTE4* |
| 51,039 | 5 | 201,207,792 | A/G | 458/39 | 7.84 × 10^-5^ | *ZmVTE4* |
| 51,038 | 5 | 201,211,449 | T/G | 24/456 | 6.44 × 10^-4^ | *ZmVTE4* |
| 51,046 | 5 | 201,222,026 | T/C | 40/440 | 7.84 × 10^-5^ | *ZmVTE4* |
| 51,045 | 5 | 201,222,043 | A/G | 39/456 | 8.06 × 10^-5^ | *ZmVTE4* |
| 50,039 | 5 | 201,276,495 | A/G | 104/383 | 2.09 × 10^-2^ | *ZmVTE4* |
| 25,857 | 5 | 202,616,083 | A/G | 139/353 | 1.28 × 10^-2^ | *ZmVTE4* |
| δ-tocopherol | | | | | | |
| 10,638 | 2 | 86,253,631 | A/G | 43/435 | 6.33 × 10^-3^ |  |
| 48,923 | 3 | 225,227,814 | A/G | 36/474 | 1.97 × 10^-2^ |  |
| 18,695 | 4 | 61,876,947 | A/G | 27/484 | 1.27 × 10^-2^ |  |
| 24,089 | 5 | 103,306,223 | A/G | 461/37 | 6.33 × 10^-3^ |  |
| 2,725 | 9 | 94,285,743 | A/G | 46/454 | 4.38 × 10^-2^ |  |
| γ-tocopherol | | | | | | |
| 7,874 | 1 | 227,897,687 | T/C | 31/479 | 3.96 × 10^-3^ |  |
| 51,195 | 3 | 213,191,036 | T/G | 33/477 | 2.83 × 10^-2^ |  |
| 18,241 | 4 | 34,181,986 | A/G | 91/402 | 2.88 × 10^-2^ |  |
| Total tocopherol | | | | | | |
| 7,874 | 1 | 227,897,687 | T/C | 31/479 | 2.45 × 10^-2^ |  |
| 11,758 | 2 | 143,273,942 | A/G | 31/444 | 4.81 × 10^-2^ |  |
| 51,195 | 3 | 213,191,036 | T/G | 33/477 | 2.45 × 10^-2^ |  |
| 18,241 | 4 | 34,181,986 | A/G | 91/402 | 2.45 × 10^-2^ |  |
| 25,291 | 5 | 171,214,128 | A/G | 465/43 | 4.11 × 10^-2^ |  |
| 32,502 | 8 | 22,962,134 | A/G | 38/442 | 2.53 × 10^-2^ |  |

^a^ The SNP code from Illumina MaizeSNP50 BeadChip. The corresponding name and source sequences of the SNPs can be obtained from the Illumina website (Illumina). SNPs with a false discovery rate less than 0.05 in the association panel of 513 lines were reported in this table. ^b^ The favorable allele is underlined. ^c^ Candidate genes were based on Figure 1, gene located within 5 Mb upstream or downstream of a SNP was considered to be a candidate gene for that SNP.
