# Supplementary material for: Genome-Wide Association Studies Identified Three Independent Polymorphisms Associated with α-Tocopherol Content in Maize Kernels
Source: PLoS One. 2012 May 15;7(5):e36807. doi: 10.1371/journal.pone.0036807 (PMC3352922; doi:10.1371/journal.pone.0036807)
Supplement: Table S3 — Summary of association results before and after excluding high-oil lines. aThe SNP code from Illumina MaizeSNP50 BeadChip. The corresponding name and source sequences of the SNPs can be obtained from the Illumina website (Illumina). SNPs with a significance level less than 1.02×10^−6^ in the association panel of 513 lines were reported in this table. bThe favorable allele is underlined. c SNPs that were significant at P<1.02×10^−6^ in CAM478 are in bold red font. NA, not available because the minor allele frequency was less than 0.05. (DOCX) [file pone.0036807.s010.docx]

**Table S3. Summary of association results before and after excluding high-oil lines**

| SNP^a^ | Chromosome | Position | Allele^b^ | Frequency in 513 lines | *P* value before excluding high-oil lines (513 lines) | Frequency in 478 lines | *P* value after excluding high-oil lines (478 lines)^c^ |
| --- | --- | --- | --- | --- | --- | --- | --- |
| α-tocopherol | | | | | | | |
| 25,801 | 5 | 198,800,752 | A/G | 439/60 | 3.59 × 10^−12^ | 409/55 | **4.50 × 10^−13^** |
| 25,815 | 5 | 199,442,506 | T/C | 257/221 | 1.60 × 10^−15^ | 238/206 | **4.83 × 10^−14^** |
| 25,817 | 5 | 199,461,718 | A/G | 355/125 | 1.09 × 10^−11^ | 335/111 | **1.45 × 10^−9^** |
| 3,462 | 5 | 199,528,414 | A/G | 99/399 | 8.85 × 10^−22^ | 97/366 | **5.59 × 10^−25^** |
| 25,820 | 5 | 199,530,006 | T/C | 95/397 | 1.77 × 10^−19^ | 93/365 | **3.81 × 10^−22^** |
| 25,821 | 5 | 199,530,947 | A/G | 266/207 | 7.10 × 10^−11^ | 250/194 | **8.57 × 10^−11^** |
| 408 | 5 | 199,970,863 | A/G | 216/263 | 9.56 × 10^−7^ | 203/241 | 1.42 × 10^−4^ |
| 53,345 | 5 | 200,017,666 | T/C | 418/71 | 3.79 × 10^−9^ | 389/66 | **3.01 × 10^−9^** |
| 25,826 | 5 | 200,023,434 | T/G | 232/248 | 7.16 × 10^−8^ | 212/234 | 3.02 × 10^−6^ |
| 51,039 | 5 | 201,207,792 | A/G | 458/39 | 1.44 × 10^−8^ | 427/35 | **8.73 × 10^−7^** |
| 51,038 | 5 | 201,211,449 | T/G | 24/456 | 1.61 × 10^−7^ | 21/425 | NA |
| 51,046 | 5 | 201,222,026 | T/C | 40/440 | 1.47 × 10^−8^ | 36/414 | 1.53 × 10^−6^ |
| 51,045 | 5 | 201,222,043 | A/G | 39/456 | 1.68 × 10^−8^ | 35/425 | **9.63 × 10^−7^** |
| δ-tocopherol | | | | | | | |
| 10,638 | 2 | 86,253,631 | A/G | 43/435 | 1.48 × 10^−7^ | 32/417 | 3.90 × 10^−3^ |
| 18,695 | 4 | 61,876,947 | A/G | 27/484 | 7.96 × 10^−7^ | 9/468 | NA |
| 24,089 | 5 | 103,306,223 | A/G | 461/37 | 2.64 × 10^−7^ | 436/27 | 1.43 × 10^−1^ |
| γ-tocopherol | | | | | | | |
| 7,874 | 1 | 227,897,687 | T/C | 31/479 | 8.26 × 10^−8^ | 16/460 | NA |
| Total tocopherol | | | | | | | |
| 51,195 | 3 | 213,191,036 | T/G | 33/477 | 5.50 × 10^−7^ | 16/460 | NA |

^a^ The SNP code from Illumina MaizeSNP50 BeadChip. The corresponding name and source sequences of the SNPs can be obtained from the Illumina website (Illumina). SNPs with a significance level less than 1.02 × 10^−6^ in the association panel of 513 lines were reported in this table. ^b^ The favorable allele is underlined. ^c^ SNPs that were significant at *P* < 1.02 × 10^−6^ in CAM478 are in bold red font. NA, not available because the minor allele frequency was less than 0.05.
