# Supplementary material for: Genome-Wide Association Studies Identified Three Independent Polymorphisms Associated with α-Tocopherol Content in Maize Kernels
Source: PLoS One. 2012 May 15;7(5):e36807. doi: 10.1371/journal.pone.0036807 (PMC3352922; doi:10.1371/journal.pone.0036807)
Supplement: Table S4 — Effect of oil content on tocopherol content. These results are based on the linear unbiased estimate of the 513 lines across three locations. a Significance level from a one-way ANOVA model, where the given compound content was the response variable and high-oil lines versus normal lines was the factor. s.d., standard deviation. (DOCX) [file pone.0036807.s011.docx]

**Table S4. Effect of oil content on tocopherol content**

| Trait | Line type | *n* | Range (μg/g) | Mean ± s.d. (μg/g) | *P* value^a^ |
| --- | --- | --- | --- | --- | --- |
| δ-tocopherol | High-oil | 32 | 0.93–8.94 | 2.07 ± 1.59 | 4.96 × 10^−8^ |
|  | Normal | 292 | 0.55–5.47 | 1.34 ± 0.53 |  |
| γ-tocopherol | High-oil | 35 | 27.52–141.25 | 79.36 ± 26.06 | 3.90 × 10^−74^ |
|  | Normal | 468 | 3.37–86.70 | 26.39 ± 12.60 |  |
| α-tocopherol | High-oil | 35 | 0.67–61.08 | 18.63 ± 13.68 | 1.28 × 10^−27^ |
|  | Normal | 466 | 0.40–31.28 | 6.73 ± 4.75 |  |
| Total tocopherol | High-oil | 35 | 50.39–198.70 | 100.36 ± 33.67 | 1.24 × 10^−88^ |
|  | Normal | 468 | 7.45–102.15 | 33.49 ± 13.18 |  |

These results are based on the linear unbiased estimate of the 513 lines across three locations. ^a^ Significance level from a one-way ANOVA model, where the given compound content was the response variable and high-oil lines versus normal lines was the factor. s.d., standard deviation.
