# Supplementary material for: Genome-Wide Association Studies Identified Three Independent Polymorphisms Associated with α-Tocopherol Content in Maize Kernels
Source: PLoS One. 2012 May 15;7(5):e36807. doi: 10.1371/journal.pone.0036807 (PMC3352922; doi:10.1371/journal.pone.0036807)
Supplement: Table S5 — Association results for two polymorphisms from ZmVTE4 with α-tocopherol in a subpanel of 155 lines. aThe favorable allele is underlined. InDel7 had three alleles, 0/4/7, with allele4 present in only four lines, allele4 and allele7 were combined because both alleles were unfavorable. 0, 0-bp insertion; 4, 4-bp insertion; 7, 7-bp insertion; 118, 118-bp insertion. bThe best linear unbiased prediction (BLUP) of each line across the two environments (Beijing 2006 and Beijing 2007) was calculated using a model in which the compound content was the responsive variable and the line was a random effect (see details in Methods). This model was fitted using the MIXED procedure in SAS. cThe phenotypic variation for αT explained by each polymorphism in each environment was calculated using the “Q + K” model in TASSEL. dAllele frequency in the whole subpanel. e Allele frequency in a subpanel excluding high-oil lines. n.d., not detected; n.s., not significant; αT, α-tocopherol; γT, γ-tocopherol. (DOCX) [file pone.0036807.s012.docx]

**Table S5. Association results for two polymorphisms from *ZmVTE4* with α-tocopherol in a subpanel of 155 lines**

| Polymorphism | Location | Allele^a^ | Frequency | Environment^b^ | *P* value (αT) | *P* value (γT) | *P* value (αT/γT) | *R^2^* (αT, %)^c^ |
| --- | --- | --- | --- | --- | --- | --- | --- | --- |
| InDel7 | 5' UTR | 0/7 | 95/41^d^ | Beijing 2006 | 1.85 × 10^−7^ | 4.32 × 10^−2^ | 3.99 × 10^−9^ | 22.67 |
|  |  |  |  | Beijing 2007 | 3.60 × 10^−7^ | 1.05 × 10^−5^ | 4.87 × 10^−10^ | 18.43 |
|  |  |  |  | BLUP | 1.70 × 10^−9^ | 4.79 × 10^−4^ | 1.53 × 10^−10^ | 23.75 |
|  |  |  | 74/30^e^ | Beijing 2006 | 1.57 × 10^−9^ | 1.10 × 10^−2^ | 9.17 × 10^−9^ | 32.93 |
|  |  |  |  | Beijing 2007 | 8.84 × 10^−13^ | 7.78 × 10^−4^ | 1.72 × 10^−9^ | 43.05 |
|  |  |  |  | BLUP | 1.52 × 10^−12^ | 1.70 × 10^−3^ | 6.58 × 10^−10^ | 39.87 |
| InDel118 | Promoter | 0/118 | 24/101^d^ | Beijing 2006 | n.s. | n.s. | 2.10 × 10^−3^ | n.d. |
|  |  |  |  | Beijing 2007 | 1.05 × 10^−2^ | n.s. | 1.51 × 10^−4^ | 5.65 |
|  |  |  |  | BLUP | n.s. | n.s. | 2.75 × 10^−4^ | n.d. |
|  |  |  | 17/75^e^ | Beijing 2006 | 3.33 × 10^−4^ | n.s. | 1.50 × 10^−5^ | 15.18 |
|  |  |  |  | Beijing 2007 | 1.10 × 10^−3^ | 3.62 × 10^−2^ | 6.03 × 10^−7^ | 13.09 |
|  |  |  |  | BLUP | 4.85 × 10^−4^ | 2.43 × 10^−2^ | 1.80 × 10^−6^ | 13.54 |

^a^ The favorable allele is underlined. InDel7 had three alleles, 0/4/7, with allele4 present in only four lines, allele4 and allele7 were combined because both alleles were unfavorable. 0, 0-bp insertion; 4, 4-bp insertion; 7, 7-bp insertion; 118, 118-bp insertion. ^b^ The best linear unbiased prediction (BLUP) of each line across the two environments (Beijing 2006 and Beijing 2007) was calculated using a model in which the compound content was the responsive variable and the line was a random effect (see details in Methods). This model was fitted using the MIXED procedure in SAS. ^c^ The phenotypic variation for αT explained by each polymorphism in each environment was calculated using the “Q + K” model in TASSEL. ^d^ Allele frequency in the whole subpanel. ^e^ Allele frequency in a subpanel excluding high-oil lines. n.d., not detected; n.s., not significant; αT, α-tocopherol; γT, γ-tocopherol.
