# Supplementary material for: Genome-Wide Association Studies Identified Three Independent Polymorphisms Associated with α-Tocopherol Content in Maize Kernels
Source: PLoS One. 2012 May 15;7(5):e36807. doi: 10.1371/journal.pone.0036807 (PMC3352922; doi:10.1371/journal.pone.0036807)
Supplement: Table S6 — Association of four polymorphisms with tocopherol-related traits across three environments in CAM478. The P values are from a “Q + K” model fitted in TASSEL. HN, Hainan; SC, Sichuan; YN, Yunnan; n.s., not significant. (DOCX) [file pone.0036807.s013.docx]

**Table S6. Association of four polymorphisms with tocopherol-related traits across three environments in CAM478**

| Locus | α-tocopherol | | |  | γ-tocopherol | | |  | α-tocopherol/γ-tocopherol | | |
| --- | --- | --- | --- | --- | --- | --- | --- | --- | --- | --- | --- |
|  | *P* (SC) | *P* (YN) | *P* (HN) |  | *P* (SC) | *P* (YN) | *P* (HN) |  | *P* (SC) | *P* (YN) | *P* (HN) |
| InDel7 | 1.2 × 10^−21^ | 1.9 × 10^−13^ | 5.7 × 10^−27^ |  | 1.9 × 10^−2^ | 1.7 × 10^−3^ | 1.2 × 10^−3^ |  | 1.3 × 10^−12^ | 9.8 × 10^−6^ | 2.7 × 10^−13^ |
| InDel118 | 6.8 × 10^−14^ | 3.4 × 10^−12^ | 1.6 × 10^−20^ |  | 1.4 × 10^−3^ | 7.5 × 10^−4^ | 3.8 × 10^−3^ |  | 1.1 × 10^−18^ | 1.6 × 10^−12^ | 6.9 × 10^−19^ |
| SNP25801 | 2.8 × 10^−5^ | 7.4 × 10^−6^ | 4.8 × 10^−12^ |  | n.s. | n.s. | 2.0 × 10^−2^ |  | 3.3 × 10^−5^ | 5.5 × 10^−3^ | 2.0 × 10^−7^ |
| SNP25815 | 2.9 × 10^−9^ | 3.9 × 10^−8^ | 5.3 × 10^−13^ |  | n.s. | 8.0 × 10^−3^ | 3.2 × 10^−3^ |  | 2.9 × 10^−9^ | 5.6 × 10^−7^ | 9.5 × 10^−8^ |

The *P* values are from a “Q + K” model fitted in TASSEL. HN, Hainan; SC, Sichuan; YN, Yunnan; n.s., not significant.
