# Supplementary material for: Genome-Wide Association Studies Identified Three Independent Polymorphisms Associated with α-Tocopherol Content in Maize Kernels
Source: PLoS One. 2012 May 15;7(5):e36807. doi: 10.1371/journal.pone.0036807 (PMC3352922; doi:10.1371/journal.pone.0036807)
Supplement: Table S7 — Evaluation of the genetic effect of four polymorphisms on tocopherol traits in F2∶3 populations. a n is the number of F2 individuals. A one-way ANOVA model with the compound as the response variable and the segregating polymorphism as the factor was used to get the significance level and phenotypic variation explained (R 2). αT, α-tocopherol; γT, γ-tocopherol; TT, total tocopherol; ND, not detected; N.P., not polymorphic; n.s., not significant. The favorable allele is underlined in each population. (DOCX) [file pone.0036807.s014.docx]

**Table S7. Evaluation of the genetic effect of four polymorphisms on tocopherol traits in F_2:3_ populations**

| Populations | InDel7 | InDel118 | SNP25801 | SNP25815 | *n*^a^ | γT (μg/g) | TT (μg/g) | αT/γT |
| --- | --- | --- | --- | --- | --- | --- | --- | --- |
| K22/Dan340 | 0/0 | N.P. | N.P. | N.P. | 40 | 16.2 ± 7.5 | 25.9 ± 8.8 | 0.74 ± 0.49 |
|  | 0/7 |  |  |  | 120 | 21.7 ± 9.8 | 28.6 ± 10.6 | 0.39 ± 0.23 |
|  | 7/7 |  |  |  | 45 | 29.6 ± 14.0 | 34.1 ± 14.4 | 0.19 ± 0.23 |
|  |  |  |  |  | *P* value | 8.4 × 10^−8^ | 2.3 × 10^−3^ | 7.9× 10^−15^ |
|  |  |  |  |  | *R^2^* | 14.9% | 5.8% | 27.5% |
| CI7/K22 | N.P. | 0/0 | N.P. | N.P. | 54 | 10.5 ± 5.8 | 20.6 ± 7.0 | 1.27 ± 0.62 |
|  |  | 0/118 |  |  | 101 | 10.3 ± 5.4 | 18.6 ± 7.1 | 0.97 ± 0.46 |
|  |  | 118/118 |  |  | 61 | 12.3 ± 7.6 | 18.1 ± 9.0 | 0.61 ± 0.38 |
|  |  |  |  |  | *P* value | n.s. | n.s. | 6.5 × 10^−11^ |
|  |  |  |  |  | *R^2^* | ND | ND | 19.8% |
| DE.EX/CI7 | N.P. | N.P. | G/G | N.P. | 22 | 18.9 ± 9.3 | 27.4 ± 11.3 | 0.45 ± 0.23 |
|  |  |  | G/A |  | 41 | 19.8 ± 6.7 | 28.7 ± 9.1 | 0.39 ± 0.19 |
|  |  |  | A/A |  | 24 | 22.5 ± 6.6 | 31.8 ± 9 | 0.37 ± 0.14 |
|  |  |  |  |  | *P* value | 0.22 | 0.28 | 0.34 |
|  |  |  |  |  | *R^2^* | 3.6% | 3.0% | 2.5% |
| 81162/Chang7-2 | N.P. | N.P. | N.P. | T/T | 40 | 27.0 ± 6.3 | 36.7 ± 7.8 | 0.27 ± 0.07 |
|  |  |  |  | T/C | 60 | 24.2 ± 5.6 | 32.2 ± 6.8 | 0.24 ± 0.08 |
|  |  |  |  | C/C | 29 | 24.4 ± 5.0 | 30.5 ± 6.0 | 0.16 ± 0.07 |
|  |  |  |  |  | *P* value | 4.6 x 10^−2^ | 8.0 x 10^−4^ | 9.4 x 10^−8^ |
|  |  |  |  |  | *R^2^* | 4.8% | 11.2% | 23.1% |

^a^ *n* is the number of F_2_ individuals. A one-way ANOVA model with the compound as the response variable and the segregating polymorphism as the factor was used to get the significance level and phenotypic variation explained (*R^2^*). αT, α-tocopherol; γT, γ-tocopherol; TT, total tocopherol; ND, not detected; N.P., not polymorphic; n.s., not significant. The favorable allele is underlined in each population.
