# Supplementary material for: Genome-Wide Association Studies Identified Three Independent Polymorphisms Associated with α-Tocopherol Content in Maize Kernels
Source: PLoS One. 2012 May 15;7(5):e36807. doi: 10.1371/journal.pone.0036807 (PMC3352922; doi:10.1371/journal.pone.0036807)
Supplement: Table S8 — Inbred lines used for expression analysis. The phenotypic data for α-tocopherol is based on the field experiment with the 155 lines in 2006 and 2007. Presence (√) and absence (NA, not available) are indicated for ZmVTE4 expression data. 2008SZ, Shangzhuang 2008; 2009CP, Changping 2009. (DOCX) [file pone.0036807.s015.docx]

**Table S8. Inbred lines used for expression analysis**

| Lines | InDel7 | InDel118 | α-tocopherol (μg/g) | 2009CP embryo | 2008SZ embryo | Seedling leaf | Seedling root | Endosperm |
| --- | --- | --- | --- | --- | --- | --- | --- | --- |
| 8701 | 7 | 118 | 3.68 | √ | √ | √ | √ | √ |
| B73 | 0 | 0 | 6.81 | √ | √ | √ | √ | √ |
| By4944 | 7 | 118 | 8.98 | √ | √ | √ | √ | √ |
| By4960 | 4 | 0 | 26.33 | NA | √ | √ | √ | √ |
| By804 | 4 | 0 | 37.73 | √ | √ | NA | NA | √ |
| By807 | 4 | 0 | 60.24 | √ | √ | √ | √ | NA |
| By843 | 0 | 118 | 63.87 | √ | √ | √ | √ | √ |
| Dan599 | 7 | 118 | 12.90 | √ | NA | √ | √ | NA |
| Gy1007 | 4 | 0 | 19.33 | √ | NA | √ | √ | NA |
| Gy462 | 0 | 118 | 19.58 | √ | √ | √ | √ | √ |
| Gy923 | 0 | 118 | 15.36 | √ | √ | √ | √ | √ |
| Hai014 | 0 | 118 | 6.85 | √ | √ | NA | NA | NA |
| Hu803 | 0 | 0 | 26.31 | √ | √ | √ | √ | √ |
| HuangC | 0 | 0 | 23.81 | √ | √ | √ | √ | NA |
| Mo113 | 7 | 118 | 1.52 | √ | √ | √ | √ | √ |
| Mo17 | 0 | 118 | 12.13 | √ | √ | √ | √ | √ |
| Qi205 | 0 | 0 | 11.85 | √ | √ | √ | √ | √ |
| Ry713 | 0 | 118 | 13.42 | √ | √ | √ | √ | √ |
| Ry732 | 7 | 118 | 0.58 | √ | √ | √ | √ | √ |
| Shen5003 | 7 | 118 | 3.85 | √ | √ | NA | NA | √ |
| Sy999 | 7 | 118 | 2.18 | √ | √ | NA | NA | √ |
| Wu109 | 0 | 118 | 6.99 | √ | √ | √ | √ | √ |
| Xi502 | 0 | 118 | 10.95 | √ | √ | √ | √ | √ |
| Ye107 | 7 | 118 | 1.10 | NA | √ | √ | √ | √ |
| Ye478 | 7 | 118 | 5.22 | √ | √ | √ | √ | √ |
| Zong3 | 7 | 118 | 1.40 | √ | √ | √ | √ | √ |

The phenotypic data for α-tocopherol is based on the field experiment with the 155 lines in 2006 and 2007. Presence (√) and absence (NA, not available) are indicated for *ZmVTE4* expression data. 2008SZ, Shangzhuang 2008; 2009CP, Changping 2009.
