# Supplementary material for: Genome-Wide Association Studies Identified Three Independent Polymorphisms Associated with α-Tocopherol Content in Maize Kernels
Source: PLoS One. 2012 May 15;7(5):e36807. doi: 10.1371/journal.pone.0036807 (PMC3352922; doi:10.1371/journal.pone.0036807)
Supplement: Table S9 — Summary of correlation between expression levels of five genes from tocopherol biosynthesis pathway and tocopherol content. The embryos collected at 20 days after pollination (n = 24) were used to perform quantitative RT-PCR of each gene. The tocopherol content was averaged over two years (Beijing 2006, 2007). The CORR procedure in SAS was used to obtain the Pearson correlation coefficients (r). “–” represents negative correlation. (DOCX) [file pone.0036807.s016.docx]

**Table S9. Summary of correlation between expression levels of five genes from tocopherol biosynthesis pathway and tocopherol content**

| Gene | Items | α-tocopherol | γ-tocopherol | δ-tocopherol | Total tocopherol |
| --- | --- | --- | --- | --- | --- |
| *ZmHPPD* | r | –0.08 | 0.19 | –0.17 | 0.11 |
|  | *P* value | 0.70 | 0.37 | 0.43 | 0.61 |
| *ZmVTE1* | r | –0.21 | –0.12 | –0.08 | –0.17 |
|  | *P* value | 0.33 | 0.58 | 0.71 | 0.42 |
| *ZmVTE2* | r | –0.19 | 0.20 | 0.09 | 0.08 |
|  | *P* value | 0.39 | 0.36 | 0.66 | 0.72 |
| *ZmVTE3* | r | –0.06 | –0.03 | –0.17 | –0.05 |
|  | *P* value | 0.77 | 0.90 | 0.42 | 0.82 |
| *ZmVTE5* | r | –0.19 | –0.15 | –0.20 | –0.20 |
|  | *P* value | 0.36 | 0.48 | 0.36 |  |

The embryos collected at 20 days after pollination (*n* = 24) were used to perform quantitative RT-PCR of each gene. The tocopherol content was averaged over two years (Beijing 2006, 2007). The CORR procedure in SAS was used to obtain the Pearson correlation coefficients (r). “–” represents negative correlation.
