# Supplementary material for: Genome-Wide Association Studies Identified Three Independent Polymorphisms Associated with α-Tocopherol Content in Maize Kernels
Source: PLoS One. 2012 May 15;7(5):e36807. doi: 10.1371/journal.pone.0036807 (PMC3352922; doi:10.1371/journal.pone.0036807)
Supplement: Table S10 — Primers used in the study. (DOCX) [file pone.0036807.s017.docx]

**Table S10. Primers used in the study**

| Category | Note | Primer sequences |
| --- | --- | --- |
| *ZmVTE4* | Promoter~exon1 | 5'-AAGGATCAGCCTTCTTTTCAA-3'/5'-GGACTGGGAGCAATGGAGC-3' |
| sequencing | 5’ UTR~intron3 | 5'-TGCCGGCACCTCTACTTTAT-3'/5'-ACCGACTGTCTGTAGTGCC-3' |
|  |  | 5'-ATAACGGAGATAGCACGAAACT-3'/5'-CCAGGAAAGCAGGTGGAATA-3' |
|  |  | 5'-CGCTACCCGATTGGTTGAA-3'/5'-ACCCTTGCCGTTACCGACTG-3' |
|  | Exon3~exon4 | 5'-TCTGCAAGTTGCTGATGCTC-3'/5'-GGCTCAGTTCATCGGGCTTT-3' |
|  | Intron3~3’ flanking | 5'-GTGGCTTTCCTGAATCCTT-3'/5'-ATTGGCACGTCGAACATACA-3' |
|  |  | 5'-TGAATCCTTACCTGGCTTTG-3'/5'-TGATCCAGGAACAGCAGAAA-3' |
| *ZmVTE4* | *ZmVTE4* | 5'-GTACTACCTCCCGGACTGG-3'/5'-CTGGATCATTAGCGGCATCAC-3' |
| expression | Actin | 5'-CGATTGAGCATGGCATTGTCA-3'/5'-CCCACTAGCGTACAACGAA-3' |
| *ZmVTE4* | InDel7 (VTE4-12F/4-12R) | 5'-TGCCGGCACCTCTACTTTAT-3'/5'-AGGACTGGGAGCAATGGAG-3' |
| PCR assay | InDel118 (VTE4-22F/4-34R) | 5'-AAAGCACTTACATCATGGGAAAC-3'/5'-TTGGTGTAGCTCCGATTTGG-3' |
