# Supplementary material for: Genome-Wide Association Studies Identified Three Independent Polymorphisms Associated with α-Tocopherol Content in Maize Kernels
Source: PLoS One. 2012 May 15;7(5):e36807. doi: 10.1371/journal.pone.0036807 (PMC3352922; doi:10.1371/journal.pone.0036807)
Supplement: Table S11 — PCR reaction system and protocol for InDel7 and InDel118 of ZmVTE4 . (DOCX) [file pone.0036807.s018.docx]

**Table S11. PCR reaction system and protocol for InDel7 and InDel118 of *ZmVTE4***

| Items | InDel7 | | | InDel118 | | |
| --- | --- | --- | --- | --- | --- | --- |
| PCR reaction | DNA (10 ng/μl) | | 2 μl | DNA (10 ng/μl) | | 2 μl |
|  | 10 × PCR buffer | | 1.5 μl | 10 × PCR buffer | | 1.5 μl |
|  | dNTP (2.5 mM) | | 1.2 μl | dNTP (2.5 mM) | | 1.2 μl |
|  | PrimerF (10 μM) | | 0.6 μl | PrimerF (10 μM) | | 0.6 μl |
|  | PrimerR (10 μM) | | 0.6 μl | PrimerR (10 μM) | | 0.6 μl |
|  | Taq (5 U/μl) | | 0.15 μl | Taq (5 U/μl) | | 0.15 μl |
|  | ddH_2_O | | 8.95 μl | ddH_2_O | | 8.95 μl |
|  | Total | | 15 μl | Total | | 15 μl |
| PCR protocol | 94 °C | 3 min | 1 cycle | 94 °C | 3 min | 1 cycle |
|  | 94 °C | \| 30 s \| \| --- \| | 35 cycles | 94 °C | \| 30 s \| \| --- \| | 35 cycles |
|  | 60 °C | 50 s |  | 58 °C | 50 s |  |
|  | 72 °C | 1 min |  | 72 °C | 1 min |  |
|  | 72 °C | 10 min | 1 cycle | 72 °C | 10 min | 1 cycle |
|  | 4 °C | ∞ | 1 cycle | 4 °C | ∞ | 1 cycle |
